# The Input of Terrestrial Dissolved Organic Carbon Enhanced Bacteria Growth Efficiency on Phytoplankton-DOC and Indigenous Lake DOC: A Microcosm Study

**DOI:** 10.3390/microorganisms13092081

**Published:** 2025-09-06

**Authors:** Zong’an Jin, Huiping Zhang, Zhengwen Liu, Erik Jeppesen, Jian Gao, Yali Tang

**Affiliations:** 1 Department of Ecology and Institute of Hydrobiology, Jinan University, 601 Huangpu Avenue West, Guangzhou 510632, China; zajin180@163.com (Z.J.); zhanghh2209@163.com (H.Z.); zliu@niglas.ac.cn (Z.L.); 2 Engineering Research Center of Tropical and Subtropical Aquatic Ecological Engineering, Ministry of Education, Jinan University, 601 Huangpu Avenue West, Guangzhou 510632, China; 3 Sino-Danish Centre for Education and Research (SDC), Beijing 100049, China; ej@bios.au.dk; 4 State Key Laboratory of Lake Science and Environment, Institute of Geography and Limnology, Chinese Academy of Sciences, Nanjing 210008, China; 5 Limnology Laboratory, Department of Biology, Middle East Technical University, 06800 Ankara, Turkey; 6 Institute for Ecological Research and Pollution Control of Plateau Lakes, School of Ecology and Environmental Science, Yunnan University, Kunming 650500, China; 7 Key Laboratory of Intelligent Health Perception and Ecological Restoration of Rivers and Lakes, Ministry of Education, Hubei University of Technology, Wuhan 430070, China

**Keywords:** bacterial growth efficiently, autochthonous DOC, allochthonous DOC, metabolic strategies, stable isotope

## Abstract

As a consequence of global climate change, lakes are increasingly receiving terrestrial dissolved organic carbon (DOC), which serves as a key substrate for microbial metabolism and fuels bacterial production (BP). However, bacteria in aquatic systems play a dual role in the carbon cycle by not only incorporating DOC into their biomass but also respiring it as CO_2_ into the atmosphere (bacterial respiration, BR). As such, the estimation of bacterial growth efficiency (BGE), defined as BP/(BP + BR), is critical for understanding lake carbon dynamics and bacterial carbon processing. To investigate the effects of terrestrial organic carbon on bacterial carbon processing in lakes, we conducted a ^13^C-labeling experiment utilizing three microcosms, each filled with 0.22 μm filtered lake water inoculated with a microbial inoculum and set as follows: no extra DOC addition as a control, adding phytoplankton-derived DOC, and adding a mixture of phytoplankton-derived and terrestrial DOC. Our findings revealed that the addition of terrestrial DOC significantly enhanced both overall BGE (40.0%) and specific BGE based on phytoplankton-DOC (62.3%) and indigenous lake DOC (27.0%). Furthermore, terrestrial DOC inputs also altered bacterial carbon consumption pathways, as indicated by isotopic evidence. These results suggest that the input of terrestrial DOC may significantly affect lake DOC processing by changing the way bacteria process phytoplankton-DOC and indigenous lake DOC. This study highlights the profound influence of terrestrial DOC on lake carbon processing and suggests that terrestrial–aquatic cross-ecosystem interactions are critical for understanding lake carbon dynamics under changing climatic conditions.

## 1. Introduction

Limnology, particularly in the context of aquatic carbon cycling, initially treated lakes as isolated ecosystems. However, attention later shifted to viewing lakes as recipients of terrestrial inputs [1]. Lakes receive significant amounts of terrestrial organic carbon from adjacent land ecosystems, primarily through runoff and precipitation [1,2,3]. Besides serving as recipients of terrestrial carbon and conduits for downstream carbon transport, lakes also process large quantities of carbon internally [4,5], functioning as hotspots of carbon cycling and fluxes relative to the surrounding landscape [1,3].

Pelagic heterotrophic bacteria, mainly represented by dominant phyla such as Prote-obacteria, Actinobacteria, and Bacteroidetes, are highly abundant and taxonomically di-verse in freshwater lakes, where they play a crucial role in processing dissolved organic carbon (DOC) [6]. They convert DOC into living carbon biomass, integrating it into the food web [4,7,8,9]. Stable isotope studies have confirmed the incorporation of allochthonous DOC by these bacterial communities [10,11]. Furthermore, terrestrial DOC inputs stimulate bacterial production [8,12] and are increasingly recognized as vital subsidies supporting the functioning of aquatic food webs [4,13,14].

In addition to supporting the aquatic food web, pelagic bacteria also respire and oxidize DOC into CO_2_ [15,16]. The strategy by which bacteria utilize a given carbon source is largely influenced by its chemical characteristics [10,17]. Terrestrial DOC, derived from terrestrial plants, is typically characterized by high aromaticity, high molecular weight, and elevated carbon-to-phosphorus (C:P) ratios—especially when compared to DOC originating from phytoplankton (e.g., DOC produced by Cyanobacteria and Chlorophyta) [6,17,18,19,20]. Consequently, terrestrial DOC is often considered recalcitrant to bacterial degradation. To maintain elemental balance under carbon-rich conditions, bacteria tend to increase respiration to eliminate excess carbon [20,21], leading to lower bacterial growth efficiency (BGE). In contrast, algal DOC is generally more labile and bioavailable, supporting higher BGE and favoring bacterial biomass production [14,22].

Lakes continuously receive DOC excreted by phytoplankton, which is rapidly assimilated by prokaryotes [23]. Occasional inputs of terrestrial organic carbon via inflows may not only contribute directly to bacterial production [11,22] but may also enhance the utilization of phytoplankton-derived DOC by serving as complementary respiratory substrate and energy source [16]. In natural systems, the coexistence of highly refractory allochthonous DOC with freshly produced, labile autochthonous DOC may stimulate bacterial growth efficiency (BGE) and promote the microbial degradation of refractory DOC via the priming effect [24,25,26]. Moreover, previous studies have shown that mixing labile and refractory DOC sources increases the diversity of carbon substrates, which can trigger co-metabolism and improve bacterial DOC consumption [27] and BGE [28]. However, some studies have reported that the addition of autochthonous DOC to allochthonous DOC has no significant effect on DOC degradation [29] or BGE [30]. Previous studies have demonstrated that bacteria preferentially allocate algal-derived DOC to biosynthesis over terrestrial DOC due to its higher bioavailability and nutritional quality [18,22]. However, the input of terrestrial DOC may affect the way they formally process algal-derived DOC, and, consequently, whole in-lake carbon processing, although this preferential carbon utilization is still not elucidated.

To investigate how terrestrial DOC inputs influence bacterial processing, particularly focusing on the bacterial processing of specific DOC sources, we conducted a ^13^C-labeling experiment using three microcosms filled with 0.22 μm filtered lake water (with indigenous lake DOC) and supplemented with a microbial inoculum. The experimental treatments consisted of the following three conditions: (1) no extra DOC addition (control), (2) ^13^C-labeled algal-derived DOC (+DOC_Algal_), and (3) a mixture of ^13^C-labeled algal-derived DOC and terrestrial maize-derived DOC (+DOC_Mixed_). We hypothesized that the addition of terrestrial DOC to autochthonous DOC enhances overall bacterial growth efficiency (BGE) by promoting more efficient utilization of algal-derived DOC. The findings from this study provide insights into how terrestrial organic carbon affects lake carbon processing and underscore the importance of considering terrestrial–aquatic cross-ecosystem interactions in aquatic carbon cycle research. This investigation is particularly relevant in the context of ongoing climate change, as lakes are increasingly receiving elevated inputs of terrestrial DOC [3,31].

## 2. Materials and Methods

### 2.1. Preparation of DOC

The algae (*Chlamydomonas* sp.) were cultured in 1.5 L conical flasks containing 800 mL of sterilized water with BG11 medium, with five independent replicates. The cultures were grown until the stationary phase, after which the flasks were sealed with transparent plastic film and incubated outdoors under natural sunlight for 48 h. A total of 0.25 g ^13^C-labeled sodium bicarbonate (NaH^13^CO_3_, 98 atom % ^13^C, ISOTEC, Sigma-Aldrich, Miamisburg, OH, USA) was added evenly to each conical flask twice a day. At the end of the labeling period, the cultures were transferred into 100 mL tubes and centrifuged at 8000 rpm for 30 min. The supernatant was discarded, and the pellets were dissolved and resuspended in 50 mL of 0.9% NaCl solution. This procedure was repeated three times to avoid possible contamination with nutrients from the cultures. The algal pellets were then freeze-dried at −80 °C and thoroughly homogenized. The δ^13^C of the pellets increased to an average of 1572.20 ± 22.07‰ due to the photosynthetic uptake of H^13^CO^3−^.

Fresh maize straw was oven-dried at 60 °C and ground into fine particles (<45 μm) using a high-speed grinder. Both algal pellets and maize straw were separately suspended in ultrapure water and homogenized using a cell disruptor for 30 min. After that, algal pellet and maize straw mixtures were frozen separately at −20 °C and quickly heated to ~36 °C (three freeze–thaw cycles) [21]. For DOC leachate extraction, both algal and maize samples were separately soaked in double-distilled water at 4 °C in the dark for 48 h, and the resulting leachates were filtered through 0.22 μm pre-rinsed cellulose acetate filters and diluted to a stock concentration of approximately 180 mg C L^−1^.

### 2.2. Experimental Design

Water samples were collected from a depth of 0.5 m in the littoral zone of NanHu Lake at Jinan University Campus in July 2020. The general chemical characteristics of NanHu Lake are presented in Table S1. The samples were filtered through 0.22 μm cellulose filters to exclude bacteria and larger organisms. The microbial inoculum was obtained by filtering lake water through 1.2 μm polycarbonate membrane filters. The incubation medium was a mixture of 225 mL of 0.22 μm filtered lake water and 25 mL of inoculum [11,21]. The cultures were distributed into modified and sterilized 500 mL Erlenmeyer flasks as described by Waichman [32] and 350 mL BOD bottles (Figure S1). The entire setup took 2–3 h. Three treatments were established: a control with no DOC addition; a single-source treatment with ^13^C-labeled algal DOC (10 mg C L^−1^), and a mixed-source treatment with maize DOC (5 mg C L^−1^) and ^13^C-labeled algal DOC (5 mg C L^−1^) added to the cultures. Each treatment had four replicates. All experimental setups were wrapped in aluminum foil and incubated in the dark at a constant temperature of approximately 25 °C for 24 to 72 h.

### 2.3. Sampling

Samples for bacterial counting (20 mL-2 μm filtrate) were taken at 0, 6, 12, 24, 48, and 72 h from cultures grown in 500 mL Erlenmeyer flasks (four replicates per treatment). Subsequently, the samples were fixed with 1.2 mL formaldehyde and analyzed using flow cytometry [33]. Bacterial biomass was estimated following the method of Theil-Nielsen and Søndergaard [34] using a conversion factor of 35 fg C cell^−1^ from bacterial abundance to biomass.

Samples for the δ^13^C analysis of bacterial biomass (δ^13^C_bacterial_) (250 mL GF/F-filtrate), respiratory CO_2_ (δ^13^CO_2_) produced by bacterial respiration (20 mL gas), and dissolved in-organic carbon (DIC; 10 mL, 0.22 μm filtrate) were collected in quadruplicate. For bacterial biomass and CO_2_, samples were taken at 0 and 24 h (representing the stationary phase) from modified 500 mL Erlenmeyer flasks, and for DIC, at 0 and 48 h from 250 mL BOD bottles. Water samples for bacterial analysis were first filtered through 2 μm cellulose acetate filters and then through pre-combusted (450 °C for 4 h) Whatman GF/F filters (nominal pore size: 0.7 μm) to collect bacteria, though some ultramicrobacteria (<0.7 μm) may pass through GF/F filters [11,14]. The filters were then acid-fumed overnight with HCl and dried at 60 °C for 24 h before storing in desiccators for isotopic analysis. To measure δ^13^C_respiration_, 20 mL of gas was drawn from each incubation vessel using a syringe and transferred into gas sampling bags. The δ^13^C_respiration_ in the control group at the start of the experiment was used to correct for background levels and to calibrate the respiration-derived δ^13^C_respiration_ in the DOC addition treatments. All glass material used for incubation and sampling was acid-washed, rinsed with deionized water, and either combusted or autoclaved (121 °C, 30 min) to eliminate potential contamination.

### 2.4. Analysis of Total Carbon, Phosphorus, Total Proteins, and Total Carbohydrates

Total carbon and nitrogen contents in algal pellets and maize straw were measured using an elemental analyzer (Elementar vario EL cube, Hanau, Germany), while total phosphorus content was determined by an inductively coupled plasma spectrometer (ICP-AES, Thermo Fisher Scientific, Waltham, MA, USA) after nitric acid digestion.

The total proteins and total carbohydrates (expressed as glucose equivalents) contents of algal pellets and maize straw were determined using a bicinchoninic acid assay kit (Nanjing Jiancheng Bioengineering Institute, Nanjing, China) with a Bio-Rad enzyme labeler and by the method of DuBois et al. [35], respectively.

### 2.5. Stable Isotope Analysis

The concentration and carbon isotope composition of respiratory CO_2_ were measured using a continuous-flow GasBench peripheral coupled to an isotope ratio mass spectrometer Delta V Advantage (Thermo Finnigan, Bremen, Germany), with an analytical precision of ±0.2‰. The concentration and carbon isotope composition of DIC were determined using a continuous-flow GasBench II automated headspace sampler interfaced with the same mass spectrometer, with an analytical precision of ±0.1‰.

Bacterial biomass carbon stable isotope values were determined using an elemental analyzer with an on-line stable isotope mass spectrometer (Flash EA 1112 HT-Delta V Advantages, Thermo Fisher Scientific, Bremen, Germany), with an analytical precision of ±0.1‰.

### 2.6. Calculations

Bacterial production rate (BP) was estimated during the exponential growth phase (0–24 h) from the increase in bacterial biomass at each time point [28]. Bacterial respiration rate (BR) was estimated from the increase in DIC concentration between 0 and 48 h in BOD bottles [21]. Bacterial growth efficiencies (BGEs) were calculated from BP and BR as follows:

(1) $$BGE=\frac{BP}{BP+BR}$$

The relative proportion of maize DOC supporting total bacterial carbon consumption was estimated using a mass balance formula based on the contribution of maize DOC in bacterial biomass and respiratory CO_2_ as follows [10]:

(2) $${Consumed\;DOC}_{maize}=\frac{BP}{BP+BR}\times {Biomass}_{maize}+\frac{BR}{BP+BR}\times {Respiratory\;CO_{2}}_{maize}$$

where Consumed DOC_maize_, Biomass_maize_, and Respiratory CO_2maize_ correspond to the proportion of maize DOC consumed, used for biosynthesis and respiration by bacteria, respectively.

### 2.7. Data Analysis

The carbon contributions of maize DOC, algal DOC, and indigenous lake DOC to the biomass and respiration CO_2_ of bacterioplankton in the +DOC_Algal_ and +DOC_mixed_ treatments were calculated using a stable isotope mixing model employing the ‘simmr’ package in R [36]. The fractionation of the isotope signature of respiratory CO_2_ and biomass during respiration and biosynthesis was corrected by +0.5‰ ± 1.5‰ and +0.6‰ ± 1.4‰, respectively, according to Guillemette et al. (2016) [10].

An independent samples *t*-test was applied using the ‘stats’ package to detect differences in total carbohydrates and total proteins contents, as well as carbon-to-nitrogen (C:N) and carbon-to-phosphorus (C:P) ratios, between fresh algae and maize straw. All data met the assumptions of normality and homogeneity of variance. One-way ANOVA followed by Tukey HSD was employed to detect differences in BP, BR, BGE, and the value of δ^13^C_bacterial_ and δ^13^C_respiration_ using the ‘stats’ package. Prior to analysis, the data (e.g., δ^13^C_biomass_ and δ^13^C_respiration_) were Box-Cox transformed, if necessary, to meet the assumptions of normality (Shapiro-Wilk test) and homogeneity of variance (Levene’s test). Welch’s ANOVA followed by Games-Howell test was performed to detect the difference in BGE on specific carbon sources among the treatments using the ‘agricolae’ package in R.

A linear mixed-effects model (LMM) was used to evaluate the effects of treatment, time, and their interaction on bacterial abundance, with Erlenmeyer flask identity included as a random effect using the function lmer from the ‘lme4’ package [37]. Post hoc multiple comparisons were conducted using Tukey’s HSD method implemented in the ‘emmeans’ package [38]. The data were log10^(X+1)^-transformed to meet the assumptions of normality and homogeneity of variance of the model residuals. All data were analyzed using R 4.3.1.

## 3. Results

### 3.1. Biochemical Properties

**Figure 1:**
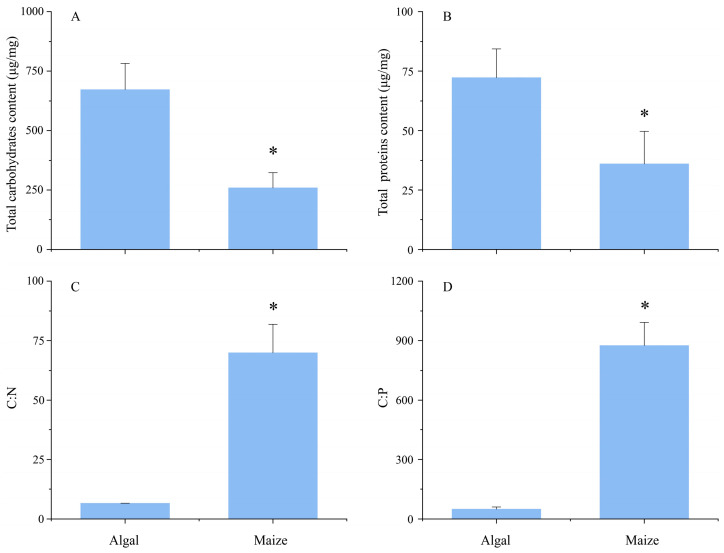
The contents of total carbohydrates (**A**), proteins (**B**), carbon-to-nitrogen (C:N) (**C**), and carbon-to-phosphorus (C:P) ratios (**D**) of fresh algal pellets and maize straw. The asterisk indicates a significant difference (*p* < 0.05) between fresh algal pellets and maize straw.

The contents of total carbohydrates and proteins were significantly higher in algal pellets than in maize straw (Table S2, Figure 1A,B). However, significantly lower C:N and C:P ratios were observed in algal pellets than in maize straw (Table S2, Figure 1C,D).

### 3.2. Bacteria Growth Curve

**Figure 2:**
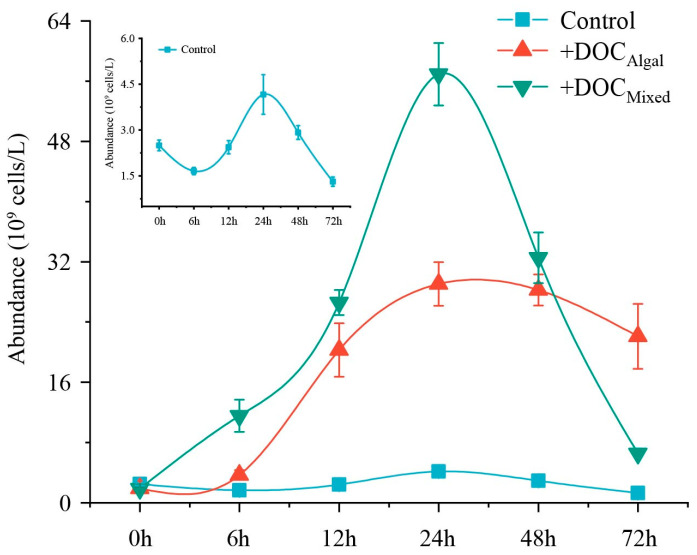
Bacterial growth in the control and +DOC_Algal_ and +DOC_mixed_ treatments during the experiment. The small panel provides an enlarged/magnified view of the control group data from the main figure.

All bacterial incubation treatments showed a logarithmic growth phase up to 24 h. followed by a gradual decrease over time (Figure 2). Treatment and time had interactive effects on bacteria abundance (Table S3). At 24 h, the abundance of bacteria was higher in the treatments with addition of extra DOC than in the control (Table S4, Figure 2). Additionally, the +DOC_Mixed_ treatment showed higher bacteria abundance than in the +DOC_Algal_ treatment at 24 h (Table S4, Figure 2).

### 3.3. Carbon Stable Isotope Compositions

**Figure 3:**
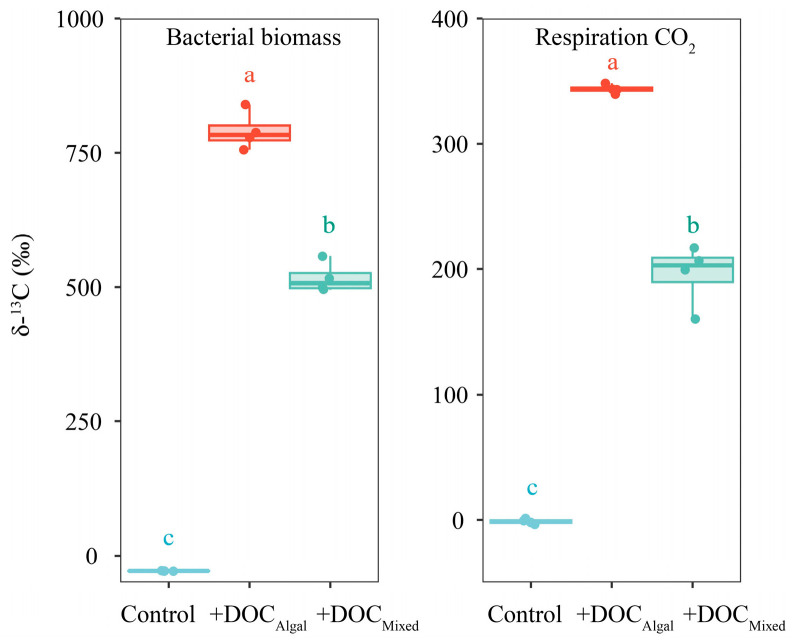
δ^13^C values of bacterial biomass and CO_2_ produced by bacterial respiration in the control, +DOC_Algal,_ and +DOC_Mixed_ treatments at 24 h. The cyan-blue, red, and cyan colors represent the δ^13^C values for the control, +DOC_Algal_, and +DOC_Mixed_ treatments, respectively. Different letters above the boxplot indicate significant differences (*p* < 0.05) among treatments.

The values of δ^13^C_bacterial_ and δ^13^C_respiration_ differed significantly among the treatments at 24 h (Table S5, Figure 3), being higher in the +DOC_Algal_ treatment (δ^13^C_bacterial_: 790.5 ± 30.5‰, δ^13^C_respiration_: 343.6 ± 3.1‰) than in the +DOC_Mixed_ treatment (δ^13^C_bacterial_: 516.7 ± 24.8‰, δ^13^C_respiration_: 195.7 ± 20.9‰) and the control (δ^13^C_bacterial_: −28.8 ± 0.2‰, δ^13^C_respiration_: −1.0 ± 1.6‰) (Table S6, Figure 3). The values of δ^13^C_bacterial_ and δ^13^C_respiration_ were also higher in the +DOC_Mixed_ treatment compared to the control (Table S6, Figure 3).

### 3.4. Bacteria Growth Efficiency on Different DOC

**Figure 4:**
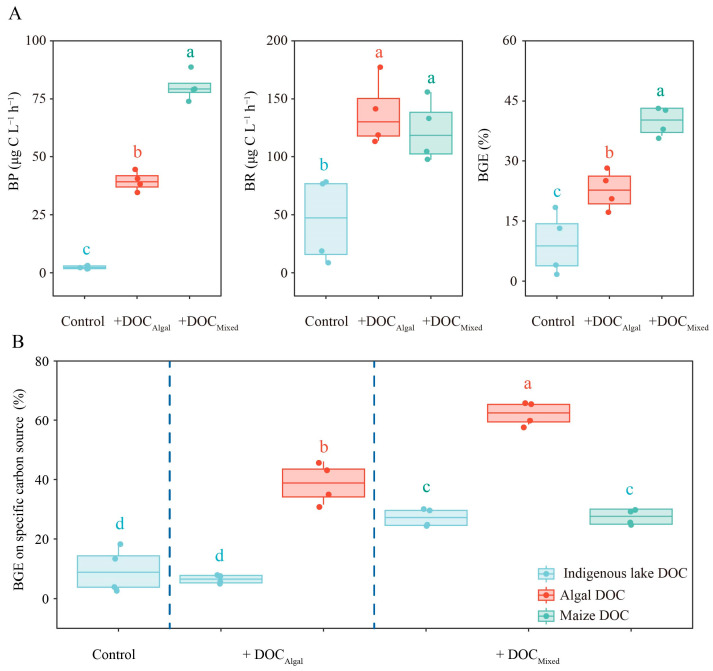
Values of bacterial respiration rate (BR), bacterial production rate (BP), bacterial growth efficiency (BGE) (**A**), and BGE on specific carbon source (**B**) in the control, +DOC_Algal_, and +DOC_Mixed_ treatments. Different letters above the boxplot indicate significant differences (*p* < 0.05) among treatments.

BP, BR, and BGE increased significantly in the DOC-supplemented treatments compared to the control (Tables S5 and S6, Figure 4A). The +DOC_Mixed_ treatment showed significantly higher BP and BGE than the +DOC_Algal_ treatment and the control, while no significant difference in BR was observed between the +DOC_Mixed_ and +DOC_Algal_ treatments (Tables S5 and S6, Figure 4A).

BGEs on specific carbon sources are presented in Figure 4B. The addition of maize DOC significantly enhanced the BGE based on algal DOC compared to the +DOC_Algal_ treatment (Tables S7 and S8). Additionally, BGE based on indigenous lake DOC was higher in the +DOC_Mixed_ treatment than in the +DOC_Algal_ treatment and the control, while there was no difference in BGE based on indigenous lake DOC between the +DOC_Algal_ treatment and the control (Tables S7 and S8).

### 3.5. Bacteria Consuming Strategy of Different DOC

**Figure 5:**
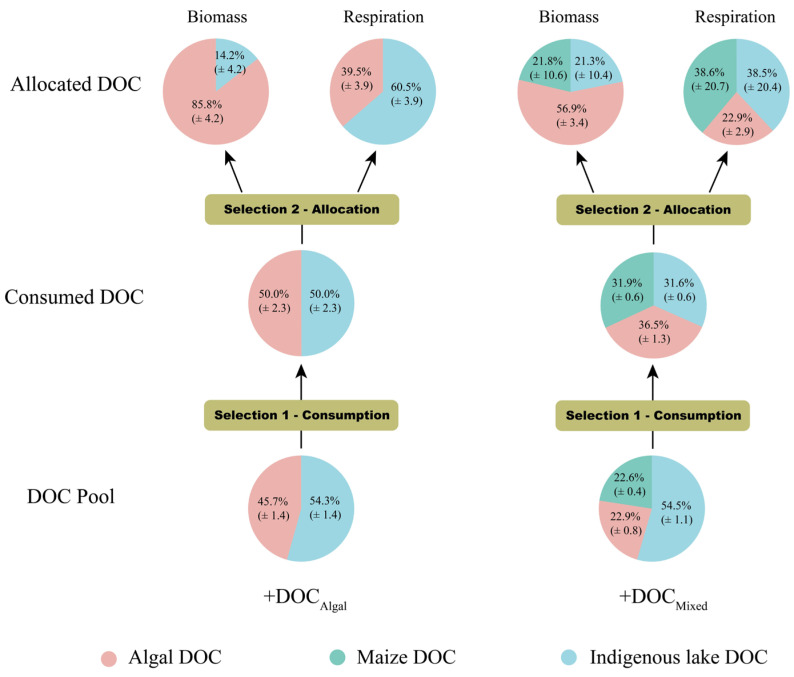
Proportions of terrestrial, algal, and indigenous lake C in the consumed DOC pool, bacterial biomass, and respiratory CO_2_. The diagram depicts the preferential consumption of algal and terrestrial DOC among DOC pools (selection 1) and the allocation of carbon sources to the respiration and growth (selection 2) of bacterioplankton. Carbon contributions of indigenous lake DOC, maize straw DOC, and algal lysate DOC to bacterioplankton biomass and respiratory CO_2_ were calculated using a carbon stable isotopic mixing model [36].

We used a mass balance to reconstruct the proportions of algal DOC, maize DOC, and indigenous lake DOC consumed by bacterial communities based on BP, BR, and the carbon contribution of three components to the biomass and respiration of bacterioplankton. Bacterial communities primarily utilized algal-derived DOC for biosynthesis, whereas the less bioavailable DOC from indigenous lake and maize straw was mainly used for respiration (Figure 5).

Bacterial communities consumed much higher proportions of algal DOC and lower proportions of indigenous lake DOC in both the +DOC_Algal_ treatment and the +DOC_Mixed_ treatment compared to the DOC pool. Interestingly, bacteria consumed much higher proportions of maize DOC in the +DOC_Mixed_ treatment compared to the DOC pool.

## 4. Discussion

By conducting a ^13^C-labeling experiment with three different microcosms, one with microbial inoculum and plain lake water as the control, one with ^13^C-labeled algal-derived DOC, and another with a mixture of ^13^C-labeled algal-derived and terrestrial macrophyte-derived DOC as treatments, we found that the addition of terrestrial DOC enhanced not only the overall BGE but also the specific BGE based on algal DOC and indigenous lake DOC. These results indicate the substantial impact of terrestrial inputs on lake carbon processing dynamics. Our findings are particularly relevant in the context of ongoing climate change, as increased frequency and intensity of precipitation and rainstorms enhance terrestrial runoff, leading to accelerated eutrophication in lakes and elevated inputs of terrestrial DOC [3,31].

The enhanced incorporation of algal DOC into bacterial biomass may result from the preferential allocation of terrestrial carbon to respiration, fulfilling the additional energy demand. This is supported by our stable isotopic labeling experiments. Although similar amounts of terrestrial and algal carbon were removed from the lake pool, 56.9% of bacterial biomass carbon originated from algal sources. In contrast, 38.6% of respired carbon and only 21.8% of assimilated carbon were derived from terrestrial carbon. Organisms synthesize biomass during anabolism using not only organic substrates but also energy [9,39]. Aerobic heterotrophs primarily obtain energy through respiration [9].

Previous studies have shown that when bacterial communities are exposed to both algal and terrestrial compounds, they preferentially utilize algal C for biomass production due to its higher availability and nutritional quality [18,22]. Therefore, the preferential incorporation of algal carbon into bacterial biomass and the differential allocation of terrestrial carbon and algal carbon might result from differences in their chemical properties and elemental stoichiometry. In terms of chemical properties, low-molecular-weight compounds are more efficiently incorporated into bacterial biomass [40]. Algal DOC is generally characterized by higher proportions of low-molecular-weight carbohydrates and lower aromaticity, making it more readily used for biosynthesis [41] and resulting in greater bacterial bioavailability and faster turnover than terrestrial DOC [6,18,19]. In contrast, the more complex and aromatic compounds in terrestrial DOC require degradation by exoenzymes before they can be utilized by bacteria [9], and the high energy demand for producing extracellular enzymes can constrain the breakdown of complex substrates [27]. The significantly higher concentrations of total carbohydrates (as glucose equivalents) in algal pellets than in maize straw further support this view. From the elemental stoichiometry perspective, algal DOC generally has a higher nutritional value (C:N ≈ 12:1) than terrestrial DOC (~50:1) [42], and such differences in nutrient composition may influence BGE, which is often inversely related to substrate C:N [43]. We did find that the BGE based on algal DOC was significantly higher than that based on maize DOC. Furthermore, bacteria have much higher specific surface-to-volume ratios, which results in greater phospholipid membrane investment per unit biomass and hence a relatively higher phosphorus demand [44,45]. This indicates a need for carbon resources with a low C:N/P ratio, as bacteria will respire the excess carbon to achieve appropriate growth [20]. A more labile carbon source can improve bacterial competitiveness for phosphorus by enhancing their uptake and sequestration capacity [46]. In our experiments, maize carbon generally had higher C:N/P ratios than algal carbon and was therefore more likely to be respired. However, other studies have shown that in lakes dominated by terrestrial carbon, bacteria tend to allocate terrestrial carbon preferentially to growth [10,47]. The authors attribute this to the selective utilization of substrates by bacterioplankton, as different bacterial taxa exhibit distinct preferences for carbon sources [10].

We also found that freshly produced maize DOC input enhanced the overall lake BGE, as well as the specific BGE based on algal DOC and indigenous lake DOC, which may be attributed to the priming effect. The priming effect is defined as the addition of labile organic matter, enhancing the utilization of refractory organic matter [24,26]. Lake indigenous DOC is what is left after fast bacteria consumption and is mainly composed of refractory carbon, with some semi-labile carbon [48,49]. The input of algal DOC is a regular and recurring process and did not significantly affect lake DOC processing in our experiment. However, the addition of freshly produced maize DOC increases substrate diversity, potentially stimulating bacterial diversity and co-metabolic activity in carbon processing [27,28], thereby enhancing BGE through the priming effect.

The specific metabolic strategies in response to different carbon sources revealed in our study may be restricted by the origin of the bacterial community. This is because community history—shaped by past environmental conditions or historical events—can influence microbial composition and determine their carbon substrate preferences [6,50]. A study based on microcosm experiments showed that when exposed to the same carbon substrate, bacteria with a terrestrial carbon history exhibited higher BR than those with an algal carbon history [6]. The authors suggested that the difference in BR was due to the fact that bacterioplankton communities with a terrestrial carbon history preferentially utilized algal DOC for respiration [10]. The bacterial community used in our study was derived from a eutrophic lake with an algal carbon history, in contrast to those shaped by terrestrial inputs [6], supporting our results of preferential use of algal DOC for biosynthesis over terrestrial DOC.

It should be noted that the results of our study stem from a short-term microcosm experiment with a simplified DOM composition, contrasting with the more complex and variable conditions of natural systems. Bacterial turnover of labile carbon happens very quickly—within hours or days [18,21]. Our results showed that bacterial abundance reached the stationary phase after 24 h of incubation, and the experiment duration was often set in the exponential growth phase in previous studies [27,28]. Despite the relatively short duration of our experiment, it clearly demonstrated the significant impact of terrestrial organic carbon inputs on lake carbon processing.

## 5. Conclusions

Our study suggests that the input of terrestrial DOC may significantly affect lake DOC processing through enhanced BGE on phytoplankton-DOC (+DOC_Mixed_ vs. +DOC_Algal_: 62.3% vs. 38.8%) due to a preferential allocation of terrestrial carbon to respiration. In addition, we also found that the addition of terrestrial DOC enhanced the BGE based on indigenous lake DOC (+DOC_Mixed_ vs. +DOC_Algal_: 27.0% vs. 6.5%). This study provides insights into how terrestrial carbon inflows influence carbon dynamics within lake ecosystems, especially in eutrophic environments.

## Data Availability

The original contributions presented in this study are included in the article/Supplementary Materials. Further inquiries can be directed to the corresponding authors.

## Supplementary Materials

The following supporting information can be downloaded at https://www.mdpi.com/article/10.3390/microorganisms13092081/s1, Figure S1: Experimental setup for determining (A) bacterial abundance/biomass, carbon stable carbon isotopes of bacterial respiration (δ^13^CO_2_), and biomass (δ^13^C_bacterial_); (B) the concentration of dissolved inorganic carbon (DIC). Left: The control treatment with no DOC addition. Middle: Single-source incubations with addition of ^13^C-algal DOC (+DOC_Algal_). Right: Mixture of maize DOC and ^13^C-algal DOC (+DOC_Mixed_). Bottom: Green line indicates the amount of maize DOC, red line shows the amount of algal DOC, and bottom white line represents the sum of added DOC. All units are in mg C L^−1^; Table S1: General physical and chemical characteristics of surface water in NanHu Lake of Jinan University Campus during experimental period; Table S2: Summary statistics of Independent Samples *t*-test for the concentrations of total carbohydrates, proteins, C:N, and C:P ratio of fresh algal and maize straw; Table S3: Type III analysis of variance (GLMM) table with Satterthwaite’s method for the growth curves of bacteria; Table S4: Summary statistics of Tukey HSD post-hoc multiple comparisons for the growth curves of bacteria; Table S5: Summary statistics of one-way ANOVA for the δ^13^C values of bacterial biomass (δ^13^C_bacterial_), CO_2_ produced by bacterial respiration (δ^13^C_respiration_), bacterial production (BP), bacterial respiration (BR) and bacterial growth efficiencies (BGE) in the control, +DOC_Algal_ and +DOC_Mixed_ treatment at 24 h; Table S6: Summary statistics of Tukey HSD post-hoc multiple comparisons for the δ^13^C values of bacterial biomass (δ^13^C_bacterial_), CO_2_ produced by bacterial respiration (δ^13^CO_2_), bacterial production (BP), bacterial respiration (BR) and bacterial growth efficiencies (BGE) in the control, +DOC_Algal_ and +DOC_Mixed_ treatment at 24 h; Table S7: Summary statistics of Welch ANOVA for the bacteria mediated allocation of BGE; Table S8: Summary statistics of Games-Howell post-hoc multiple comparisons for the bacteria mediated allocation of BGE at 24 h. MA and AA represent algal DOC in the +DOC_Mixed_ treatment and the +DOC_Algal_ treatment, respectively. MM represents maize DOC in the +DOC_Mixed_ treatment. CS, AS, and MS represent indigenous lake DOC in the Control, +DOC_Algal_ and +DOC_Mixed_ treatments, respectively. Different lowercase letters indicate a significant difference (*p* < 0.05) among treatments.

## Abbreviations

The following abbreviations are used in this manuscript:

DOC	Dissolved organic carbon
BP	Bacterial production rate
BR	Bacterial respiration rate
BGE	Bacterial growth efficiency
C:N	The ratio of carbon to nitrogen
C:P	The ratio of carbon to phosphorus
LMM	A linear mixed-effects model
δ^13^C_bacteria_	The δ^13^C value of bacterial biomass
δ^13^CO_2_	The δ^13^C value of CO_2_ produced by bacterial respiration
