# Supplementary material for: The Input of Terrestrial Dissolved Organic Carbon Enhanced Bacteria Growth Efficiency on Phytoplankton-DOC and Indigenous Lake DOC: A Microcosm Study
Source: Microorganisms. 2025 Sep 6;13(9):2081. doi: 10.3390/microorganisms13092081 (PMC12472250; doi:10.3390/microorganisms13092081)
Supplement: Supplementary file 1 [file microorganisms-13-02081-s001.zip › microorganisms-3773161-supplementary.pdf]

Supplementary Materials. Figure S1

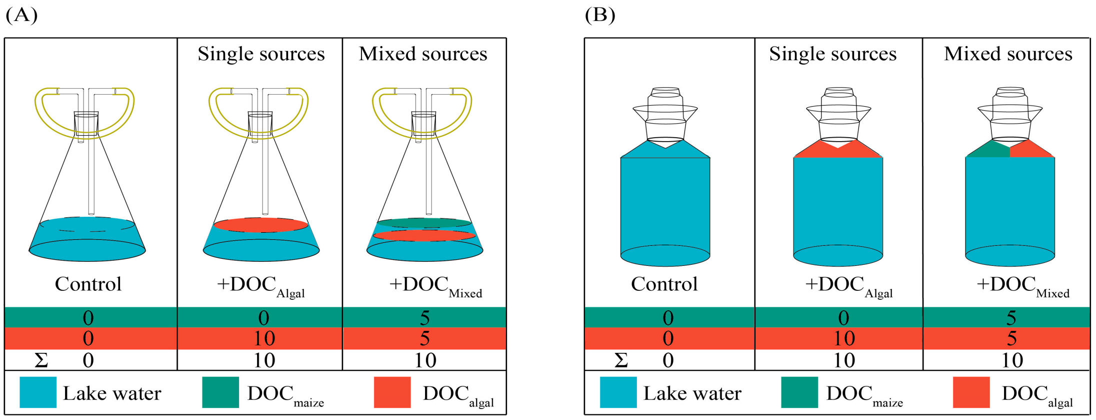

Figure S1. Experimental setup for determining (A) bacterial abundance/biomass, carbon stable carbon isotopes of bacterial respiration ($\delta^{13}CO_2$), and biomass ($\delta^{13}C_{bacterial}$); (B) the concentration of dissolved inorganic carbon (DIC). Left: The control treatment with no DOC addition. Middle: Single-source incubations with addition of ^{13}C-algal DOC (+DOC_{Algal}). Right: Mixture of maize DOC and ^{13}C-algal DOC (+DOC_{Mixed}). Bottom: Green line indicates the amount of maize DOC, red line shows the amount of algal DOC, and bottom white line represents the sum of added DOC. All units are in mg C L^{-1}.

Supplementary Materials. Table S1-S8

Table S1 General physical and chemical characteristics of surface water in NanHu Lake of Jinan University Campus during experimental period.

	**pH**	**Water depth**	**TN**	**TP**	**Chl *a***	**DOC**
		cm	mg/L	mg/L	μg/L	mg/L
NanHu Lake	6.96±0.26	138.33±15.57	1.60±0.16	0.11±0.01	99.27±8.98	14.26±0.85

Table S2 Summary statistics of Independent Samples t-test for the concentrations of total carbohydrates, proteins, C:N, and C:P ratio of fresh algal and maize straw.

	**T**	**df**	**p**
Total carbohydrates	5.656	4	0.005
Total proteins	3.435	4	0.026
C:N	-9.210	4	<0.001
C:P	-12.301	4	<0.001

Table S3 Type III analysis of variance (GLMM) table with Satterthwaite's method for the growth curves of bacteria.

	**Sum Sq**	**Mean Sq**	**NumDF**	**DenDF**	**F**	**p**
treatment	1.896	0.948	2	9	594.33	<0.001
hour	7.786	1.557	5	45	976.09	<0.001
Treatment*hour	3.848	0.385	10	45	241.24	<0.001

Table S4 Summary statistics of Tukey HSD post-hoc multiple comparisons for the growth curves of bacteria.

**hour**	**Contrast**	**estimate**	**SE**	**df**	**t.ratio**	**p**

0	Control-Algal	0.111	0.036	31.6	3.088	0.011
	Control-Mixed	0.121	0.036	31.6	3.391	0.005
	Algal-Mixed	0.011	0.036	31.6	0.304	0.951
6	Control-Algal	-0.345	0.036	31.6	-9.651	<0.001
	Control-Mixed	-0.839	0.036	31.6	-23.449	<0.001
	Algal-Mixed	-0.494	0.036	31.6	-13.798	<0.001
12	Control-Algal	-0.918	0.036	31.6	-25.649	<0.001
	Control-Mixed	-1.039	0.036	31.6	-29.036	<0.001
	Algal-Mixed	-0.121	0.036	31.6	-3.387	0.005
24	Control-Algal	-0.848	0.036	31.6	-23.688	<0.001
	Control-Mixed	-1.140	0.036	31.6	-31.870	<0.001
	Algal-Mixed	-0.293	0.036	31.6	-8.182	<0.001
48	Control-Algal	-0.987	0.036	31.6	-27.576	<0.001
	Control-Mixed	-1.047	0.036	31.6	-29.256	<0.001
	Algal-Mixed	-0.060	0.036	31.6	-1.680	0.228
72	Control-Algal	-1.224	0.036	31.6	-34.208	<0.001
	Control-Mixed	-0.703	0.036	31.6	-19.640	<0.001
	Algal-Mixed	0.521	0.036	31.6	14.568	<0.001

Table S5 Summary statistics of one-way ANOVA for the $\delta^{13}C$ values of bacterial biomass ($\delta^{13}C_{bacterial}$), CO_2 produced by bacterial respiration ($\delta^{13}C_{respiration}$), bacterial production (BP), bacterial respiration (BR) and bacterial growth efficiencies (BGE) in the control, +DOC_{Algal} and +DOC_{Mixed} treatment at 24 h.

	**Df**	**Sum Sq**	**Mean Sq**	**F**	**p**
$\delta^{13}C_{biomass}$	2	555.5	277.73	6933	<0.001
$\delta^{13}C_{respiration}$	2	2605.6	1302.8	867.4	<0.001
BP	2	12123	6062	328.6	<0.001
BR	2	19685	9843	10.09	0.005
BGE	2	0.189	0.094	30.11	<0.001

Table S6 Summary statistics of Tukey HSD post-hoc multiple comparisons for the $\delta^{13}C$ values of bacterial biomass ($\delta^{13}C_{bacterial}$), CO_2 produced by bacterial respiration ($\delta^{13}CO_2$), bacterial production (BP), bacterial respiration (BR) and bacterial growth efficiencies (BGE) in the control, +DOC_{Algal} and +DOC_{Mixed} treatment at 24 h.

	**Contrast**	**diff**	**lwr**	**upr**	**p**
$\delta^{13}C_{biomass}$	Algal-Control	15.217	14.822	15.613	<0.001
	Mixed -Control	13.492	13.097	13.887	<0.001
	Mixed-Algal	-1.725	-2.120	13.888	<0.001
$\delta^{13}C_{respiration}$	Algal-Control	34.861	32.442	37.281	<0.001
	Mixed -Control	25.533	23.113	27.953	<0.001
	Mixed-Algal	-9.328	-11.748	-6.909	<0.001
BP	Algal-Control	37.129	28.650	45.608	<0.001
	Mixed -Control	77.829	69.349	86.308	<0.001
	Mixed-Algal	40.700	32.220	49.179	<0.001
BR	Algal-Control	92.563	30.914	154.212	0.006

	Mixed -Control	77.202	15.553	138.851	0.017
	Mixed-Algal	-15.361	-77.010	46.288	0.772
BGE	Algal-Control	0.134	0.024	0.245	0.020
	Mixed -Control	0.307	0.196	0.417	<0.001
	Mixed-Algal	0.172	0.062	0.283	0.005

Table S7 Summary statistics of Welch ANOVA for the bacteria mediated allocation of BGE.

**F**	**num df**	**denom df**	**p**
129.740	5.000	8.090	<0.001

Table S8 Summary statistics of Games-Howell post-hoc multiple comparisons for the bacteria mediated allocation of BGE at 24 h. MA and AA represent algal DOC in the +DOC_{Mixed} treatment and the +DOC_{Algal} treatment, respectively. MM represents maize DOC in the +DOC_{Mixed} treatment. CS, AS, and MS represent indigenous lake DOC in the Control, +DOC_{Algal} and +DOC_{Mixed} treatments, respectively. Different lowercase letters indicate a significant difference ($p < 0.05$) among treatments.

**Treatment**	**Mean**	**Std**	**Letter**
MA	62.272	3.665	a
AA	38.833	6.708	b
MM	27.408	3.095	c
MS	27.001	3.066	c
CS	9.360	7.455	d
AS	6.537	1.712	d
